# Associations between frailty and cognitive impairment in Parkinson´s disease: a cross-sectional study

**DOI:** 10.1007/s40520-024-02922-4

**Published:** 2025-01-03

**Authors:** M. C. Sousa-Fraguas, G. Rodríguez-Fuentes, D. Lastra-Barreira, N. M. Conejo

**Affiliations:** 1 Department of Surgery and Medical-Surgical Specialties, Faculty of Medicine and Health Sciences, University of Oviedo, Oviedo, 33006 Spain; 2 Faculty of Physiotherapy, Department of Surgery and Medical-Surgical Specialties, University of Oviedo, Oviedo, E-33006 Spain; 3 Corporación Fisiogestión, Gijón, 33202 Spain; 4 Instituto de Neurociencias del Principado de Asturias (INEUROPA), University of Oviedo, Oviedo, 33003 Spain; 5 Instituto de Investigación Sanitaria del Principado de Asturias (ISPA), Oviedo, 33006 Spain; 6 Laboratory of Neuroscience, Department of Psychology, Neurocon Research Group, University of Oviedo, Oviedo, 33003 Spain; 7 Department of Functional Biology and Health Sciences, Faculty of Physiotherapy, Universidade de Vigo, Pontevedra, 36005 Spain; 8 HealthyFit Research Group, Galicia Sur Health Research Institute (IIS Galicia Sur), SERGAS-UVIGO, Vigo, Pontevedra, Spain; 9 Jove Hospital Foundation, Gijón, 33290 Spain

**Keywords:** Parkinson´s disease, Frailty, Cognitive dysfunction, Association

## Abstract

**Background:**

The presence of frailty is common in people with Parkinson’s disease, as is cognitive dysfunction. Previous research on frailty has focused on the physical aspects of the pathology.

**Aims:**

To analyze the relationship between frailty and cognitive impairment in patients with Parkinson’s disease and to know which disease characteristics are associated with frailty.

**Methods:**

An observational, correlational and cross-sectional study was conducted. Participants were recruited from a Home Rehabilitation Service and two Parkinson’s Associations. An individualized assessment was carried out by means of a structured interview. Frailty was assessed with the Fried scale and cognitive function with the Mini Mental State Examination and the Parkinson’s Disease Cognitive Rating Scale.

**Results:**

90 patients were recruited, 60% men, with a mean age of 73.50 (6.71) years. Frailty was associated with age and disease severity (*p* < 0.05). Frail patients presented worse cognitive performance relative to pre-frail and robust patients. A negative correlation (coefficient − 0.503) was observed between frailty and measures of patients’ cognitive function (*p* < 0.05).

**Discussion:**

The coexistence of frailty and cognitive impairment should be assessed, as PD patients with both conditions are more vulnerable and have a higher chance of experiencing adverse effects.

**Conclusion:**

Frail patients with Parkinson’s disease present an impairment of cognitive functions dependent on cortical and subcortical regions, being these regions more preserved in the case of robust. The development of programs for early detection of frailty and cognitive function in these patients is necessary to implement strategic intervention plans focused on reversing frailty and cognitive impairment.

**Protocol registration number:**

http://www.ClinicalTrials.gov ID: NCT05388526.

**Supplementary Information:**

The online version contains supplementary material available at 10.1007/s40520-024-02922-4.

## Introduction

Parkinson’s Disease (PD) is the second most common neurodegenerative pathology after Alzheimer’s Disease [1]. The disease is a result of the loss of dopaminergic neurons in the *pars compacta* of the substantia nigra [2]. It affects around 0.3% of the general population and 1 to 3% of the population over 65 years of age. It is expected that the number of people affected will increase from 8.7 to 9.3 million by 2030 [3]. In Europe, the prevalence data ranges from 65 to 12,500 per 100,000 inhabitants [2]. PD is characterized as a complex, heterogeneous and progressive pathology with motor and non-motor symptoms [4, 5]. The main motor symptoms are tremor, rigidity, bradykinesia and postural instability [2], which typically appear when 50 to 70% of dopaminergic neurons are affected [5]. Non-motor symptoms include autonomic dysfunction, sleep and mood disturbances, and cognitive and sensory disturbances [1]. Cognitive dysfunction is considered one of the most prevalent non-motor symptom in PD [6]. The prevalence of mild cognitive impairment in PD is 40% [7]. It can vary from mild cognitive difficulties, even at the beginning of the pathology, to the development of dementia after several years of disease evolution [8]. Its clinical manifestations can encompass different cognitive domains, such as executive function, visuospatial ability, memory and language, although it can also include attention fluctuations and visual hallucinations [9]. Cognitive impairment hinders the performance of basic activities of daily living and negatively affects the patient’s quality of life [10].

Frailty is defined as a clinical condition in which an individual shows increased vulnerability to stressors due to the impairment of various physiological systems [11]. Frail patients are more likely to develop adverse health outcomes, such as falls, fractures, need for long-term care, disability, hospitalizations, and death [12, 13]. The prevalence of frailty in older adults ranges from 12 to 24%, with pre-frailty ranging from 46 to 49%, depending on the assessment instrument used and the study population [14]. Commonly used scales to assess frailty include the frailty index and the Fried phenotype, the latter being the most broadly used [15]. This frailty phenotype is defined by the presence of three to five of the following criteria: unintentional weight loss, exhaustion, low physical activity, slow gait, and weakness [12].

The presence of frailty is common in people who suffer from PD (pwPD) [16] and is associated with both motor and non-motor symptoms of the disease [17]. The presence of frailty has been associated with higher Hoehn & Yahr (H&Y) stages, longer disease duration, more severe impact on motor and non-motor symptoms, including dementia, reduced quality of life, falls, and increased likelihood of institutionalization and mortality [18, 19]. Frailty prevalence varies from 29 to 67% in pwPD [20].

There is growing interest in the scientific community to study the relationship between frailty and cognitive function in older adults. Research in frail older adults has found them to exhibit a significantly worse performance compared to robust groups on both global and cognitive domain-specific tests of cognition [21, 22]. The association between these two concepts may be bidirectional, given that frailty increases the risk of future cognitive deterioration and cognitive deterioration increases the risk of frailty, which would indicate that frailty and cognition interact within a cycle of deterioration associated with aging [23]. The concept of cognitive frailty has been developed as a heterogeneous and reversible clinical manifestation in which physical frailty and mild cognitive impairment coexist without the presence of neurodegenerative diseases [24]. Some authors have linked the combination of frailty and cognitive impairment as a predictor of adverse outcomes among older adults [25]. The combination of frailty and cognitive impairment is associated with an increased risk of mortality, disability, hospitalization, and reduced quality of life. The combination of pre-frailty and mild cognitive impairment is linked to dementia and mortality [25].

Most of the frailty research conducted in pwPD focuses on the physical aspects of the disease, probably because PD has a similar clinical picture to physical frailty [26]. However, its non-motor symptoms should also be taken into account. Thus, although evidence on frailty is emerging in neurology [30] it remains scarce in PD [27–29] and studies on the relationship between frailty and cognition in pwPD are needed. Filling this gap could allow the development of strategic plans for prevention and treatment of both frailty and cognitive impairment. Due to the neurodegenerative condition of their pathology, pwPD are a vulnerable population. For this reason, identifying both frailty and cognition becomes necessary to try to prevent deterioration of both the physical and cognitive dimensions, as well as to identify those pwPD with a higher risk of developing dementia and thus improve the overall health and well-being of the patient.

This study hypothesizes that frail pwPD have worse cognitive performance than robust pwPD. The main objective is to analyze the relationship between frailty and cognitive impairment in pwPD and to understand what characteristics of the disease are associated with frailty.

## Materials and methods

### Study sesign

An observational, correlational, and cross-sectional study was conducted. It was registered with clinicaltrials.gov (NCT05388526) and followed the recommendations of the Strengthening the Reporting of Observational Studies in Epidemiology (STROBE) [31].

### Participants, setting and eligibility criteria

The participants in this study were recruited from a Home Rehabilitation Service and two Parkinson’s Associations in the cities of Gijón, Oviedo, Avilés, and Mieres, in the Principality of Asturias, Spain.

The inclusion criteria were: men and women with PD stages 1–3 according to the Hoehn & Yahr scale (H&Y), residents of the Principality of Asturias, a score of more than 24 points in the Mini Mental State Examination (MMSE), and providing signed consent. The exclusion criteria were: secondary or atypical parkinsonism, acute illness causing clinical instability, stages 4 and 5 on the H&Y scale, inability to speak or communicate, patients with dementia and/or in the terminal phase.

### Sample size calculation

To calculate the sample size, a confidence level of 95%, a power of 80% and an effect size of 0.38 (estimated based on initial data from our work) were considered. Taking into account a potential 15% loss, each of the three groups required a sample size of 28 participants.

### Data collection

Patient assessment, including the collection of information and the completion of the questionnaires, was carried out by two physiotherapists with experience in neurological physiotherapy, following the same guidelines and assessment criteria. The evaluations were carried out between January 2022 and June 2023 in person. An individualized assessment was carried out through a structured and personal interview at the patients’ homes or at the facilities of the Parkinson’s Associations. In order to facilitate their understanding, the language used during the interviews was adapted to the ability of the participants. Ambiguity and the use of vague terms were avoided. Patients were evaluated in the ON phase.

### Study variables

The variables collected included sociodemographic data such as gender (male and female), age (continuous and age range), social situation (including living situation, presence or absence of a caregiver), and marital status (single, married, divorced or widowed). Data on the educational level divided into four categories (no formal education, completed primary education, secondary education, and university) were also included. The body mass index (BMI) of the pwPD was calculated using a Tanita BC-731 model electronic scale to measure weight and a Seca 213 portable stadiometer with 1 mm division to measure height.

Charlson Comorbidity Index was calculated [32]. The scale consists of 19 items. Absence of comorbidity was considered between 0 and 1 points, low comorbidity 2 points, high comorbidity between 3 and 5 points, and severe comorbidity more than 5 points.

The variables linked to PD were the years of disease evolution since diagnosis and the total amount of the dopaminergic treatment, which was determined using the levodopa equivalent daily dose (LEDD) [33]. The stage of the disease was determined using the H&Y scale [34]. This is divided into 6 stages. It starts at stage 0, where no symptoms of the disease occur, and goes to stage 5, where the patient is totally dependent. In addition, the Movement Disorder Society-Unified Parkinson’s Disease Rating Scale (MDS-UPDRS) was used to evaluate the severity of the symptoms and the functional state of each patient. The values of this scale range from 0 to 265 points, a higher score implies a greater severity of the disease [35, 36].

Frailty was measured using the five criteria proposed by Fried et al. [12]. The data collected to determine the frailty score were: (1) unintentional weight loss ≥ 4.5Kg in the last year; (2) feeling of exhaustion through two questions from the Center for Epidemiologic Studies Depression Scale (CES-D) [37]; (3) low level of physical activity through kilocalorie consumption in the last week adjusted by sex; (4) gait slowness assessed through speed when walking 4.5 m, adjusted for height and sex; (5) muscle weakness assessed through maximum grip strength with the dominant hand measured with a Saehan dynameter, adjusted for sex and BMI (Detailed information in Supplementary data). Patients were classified as frail if they met three or more criteria, pre-frail if they met one or two, and robust if they did not meet any positive criteria.

Cognitive function was assessed using the MMSE, which evaluates spatial and temporal orientation, immediate memory, recall memory, attention, concentration and calculation, language, naming, repetition, reading, writing, comprehension, and constructive apraxia. The score ranges from 0 to 30 points and the cut-off point for the detection of dementia was 24 points [38].

In addition, the Parkinson´s Disease Cognitive Rating Scale (PD-CRS) was used. This scale is designed to detect the full spectrum of cognitive dysfunction that occurs in the course of PD. It comprises nine cognitive tasks distributed into two subscores, with a maximum score of 134 points: fronto-subcortical (verbal fixation memory, 12 points; sustained attention, 10 points; working memory, 10 points; drawing a clock, 10 points; delayed verbal memory, 12 points; alternating verbal fluency, 20 points; verbal action fluency, 30 points) and posterior cortical (confrontational naming, 20 points; and copying a clock, 10 points). The cut-off points for detection of mild cognitive impairment and dementia were ≤ 81 and ≤ 64, respectively [39].

### Ethical considerations

This study was approved by the Research Ethics Committee of the Principality of Asturias (code CEImPA 2022 − 195) and all participants signed an informed consent.

### Statistical methods

A descriptive analysis of each collected variable was performed, providing position measures such as mean and median, and dispersion measures such as standard deviation for quantitative variables, and absolute and relative frequency distributions for qualitative ones.

The relationships between qualitative variables were studied with the Pearson Chi Square test or the Fisher test, depending on whether or not the hypothesis about expected frequencies was met. The differences between quantitative variables between three groups were evaluated using the ANOVA test or the Kruskal-Wallis test after verification of the hypotheses of normality and/or homoscedasticity.

Correlations were assessed with the Pearson or Spearman correlation coefficient and test, depending on whether or not the normality hypothesis was met. Finally, a multinomial regression model was built to determine the factors associated with frailty, providing the corresponding odds ratio, their 95% confidence intervals and the associated significance for each of the two equations (pre-frail vs. robust and frail vs. robust). Subsequently, goodness was evaluated with the likelihood ratio test and the Nagelkerke’s R2 was calculated.

The significance level used was 0.05. Statistical analyses were performed using the R program (R Development Core Team) version 4.1.3.

## Results

The final sample was composed of 90 pwPD. Table 1 shows the sociodemographic and clinical characteristics of the total sample. A total of 60% were men, with an average age of 73.50 (6.71) years. A total of 84.4% lived with their family and 67.8% had a caregiver. Most frequent comorbidities were heart failure (14.4%), chronic respiratory disease (12.2%), diabetes (12.2%), and cerebrovascular disease (10%).

**Table 1:** Sociodemographic and clinical characteristics

Patient characteristics	Total (*n* = 90)
Range age years	
50–59, n (%)	4 (4.4%)
60–69, n (%)	16 (17.8%)
70–79, n (%)	52 (57.8%)
> 80, n (%)	18 (20%)
Sex	
Male, n (%)	54 (60%)
Female, n (%)	36 (40%)
Level of education	
No education, n (%)	23 (25.6%)
Completed primary education, n (%)	28 (31.1%)
Secondary education, n (%)	24 (26.7%)
University, n (%)	15 (16.7%)
Marital status	
Single, n (%)	4 (4.4%)
Married, n (%)	69 (76.7%)
Divorced, n (%)	3 (3.3%)
Widowed, n (%)	14 (15.6%)
Charlson index, mean (SD)	0.58 (0.82)
Disease duration (years), mean (SD)	7.83 (6.28)
Hoenh & Yarh, mean (SD)	2.01 (0.71)
Body mass index, mean (SD)	27.55 (4.87)
LEDD, mean (SD)	862.26 (500.15)
MDS-UPDRS Total, mean (SD)	52.83 (20.93)
MDS-UPDRS part I, mean (SD)	14.22 (6.25)
MDS-UPDRS part II, mean (SD)	15.92 (6.86)
MDS-UPDRS part III, mean (SD)	18.97 (9.82)
MDS-UPDRS part IV, mean (SD)	3.72 (3.56)
MMSE, mean (SD)	27.64 (1.67)
PD-CRS total, mean (SD)	74.19 (18.87)
PD-CRS frontal-subcortical, mean (SD)	49.09 (16.25)
PD-CRS posterior-cortical, mean (SD)	25.10 (3.61)

*Abbreviations* LEDD Levodopa Equivalent Daily Dose; MDS-UPDRS Movement Disorder Society-Unified Parkinson´s Disease Rating Scale; MMSE mini mental state examination; PD-CRS Parkinson’s Disease Cognitive Rating Scale; SD standard deviation

Table 2 shows the sociodemographic and clinical characteristics of the participants according to frailty status. The results showed that age and age range were significantly related to frailty (F(2.87) = 3.91, *p* = 0.02). Frail people were concentrated in the equal to or greater than 80 years age group with statistically significant differences compared to younger groups. The distribution between men and women and the duration in years of the disease since diagnosis were not relevant in the detection of frailty (χ²(2, 90) = 3.30, *p* = 0.19). There were no statistically significant differences in the Charlson index among the three groups (χ²(2) = 4.03, *p* = 0.14). Regarding the specific categories of this index, it was observed that the proportion of patients without comorbidities was similar across the three frailty groups. However, the proportions of patients with low comorbidity varied between the groups, with a higher presence in the pre-frail and frail groups, although these differences were not statistically significant (χ²(4, 90) = 5.05, *p* = 0.21). Regarding the severity of the disease, both on the H&Y scale and on the MDS-UPDRS scale, frail patients had higher scores with greater severity and impact of the disease, with statistically significant differences (χ²(2) = 23.89, *p* < 0.001, F(2.87) = 24.85, *p* < 0.001, respectively). In turn, these frail patients obtained lower scores on cognitive impairment assessment scales. However, robust patients, compared to frail patients, obtained higher scores, for those with preserved fronto-subcortical and posterior regions. Based on the data collected from the total PD-CRS scale, pre-frail patients fall between robust and frail patients, with statistically significant differences among the three groups (χ²(2) = 22.11, *p* < 0.001). When analyzing the differences between the frontal-subcortical and posterior-cortical cognitive profiles in relation to frailty status, it was observed that the frontal-subcortical score decreased significantly with the increase in frailty, being more pronounced in the frail group in comparison to the rest of the groups (χ²(2) = 20.42, *p* < 0,001). The differences in posterior cortical scores were also statistically significant, but less marked between the pre-fragile and fragile group (F(2.87) = 12.06, *p* < 0,001). This indicates that frailty is linked to cognitive impairment that differently impacts frontal-subcortical functions and posterior cortical functions.

**Table 2:** Sociodemographic and clinical characteristics of the participants by Frailty Status

Patient characteristics	Robust (*n* = 29)	Pre-frail (*n* = 33)	Frailty (*n* = 28)	*p*-value
Age, mean (SD)	71.75 (6.18)^a^	73.03 (6.95) ^a, b^	76.18 (6.27)^b^	***p*** ** = 0.024**
Age group				***p*** ** = 0.022**
50–59, n (%)	2 (50%)	2 (50%)	0 (0%)	
60–69, n (%)	9 (56.25%)	4 (25%)	3 (18.75%)	
70–79, n (%)	16 (30.77%)	22 (42.31%)	14 (26.92%)	
> 80, n (%)	2 (11.11%)	5 (27.78%)	11 (61.11%)	
Sex				*p* = 0.192
Male, n (%)	20 (37.04%)	21 (38.89%)	13 (24.07%)	
Female, n (%)	9 (25%)	12 (33.33%)	15 (41.67%)	
Disease duration (years), Me (IQR)	6 (2–9)	7 (4–11)	7.50 (4–10)	*p* = 0.39
Hoenh & Yarh, Me (IQR)	1.50 (1–2)	2 (1.50–2.50)	2.50 (2.50-3)	***p*** ** < 0.001**
Body mass index, mean (SD)	27.04 (4.03)	27.81 (4.77)	27.78 (5.82)	*p* = 792
LEDD, Me (IQR)	660 (475-958.75)	941.50 (587.50–1285)	748.50 (428.91-1130.94)	*p* = 164
MDS-UPDRS Total, mean (SD)	36.55 (13.11)	54.30 (19.04)	67.96 (17.63)	***p*** < 0.001
MDS-UPDRS part I, Me (IQR)	9 (6–13)^a^	16 (12–18)^b^	17 (14–19)^b^	***p*** ** < 0.001**
MDS-UPDRS part II, mean (SD)	11.31 (4.81)	15.76 (6.11)	20.89 (6.18)	***p*** ** < 0.001**
MDS-UPDRS part III, Me (IQR)	12 (9–15)	17 (13–24)	25.50 (19.75–33.25)	***p*** ** < 0.001**
MDS-UPDRS part IV, Me (IQR)	2 (0–5)	5 (3–7)	1.50 (0–7)	*p* = 0.082
MMSE, Me (IQR)	28 (28–29)^a^	28 (27–29)^a, b^	27 (25-28.25)^b^	***p*** ** = 0.018**
PD-CRS total, Me (IQR)	87 (81–98)	71 (65–82)	61 (51.50–70.50)	***p*** ** < 0.001**
PD-CRS frontal-subcortical, Me (IQR)	59 (55–70)^a^	44 (40–57)^b^	38 (30.75-45)^a, b^	***p*** ** < 0.001**
PD-CRS posterior-cortical, mean (SD)	26.93 (2.37)^a^	22.45 (3.54)^a^	22.79 (3.59)^b^	***p*** ** < 0.001**

*Abbreviations* IQR interquartile range; LEDD Levodopa Equivalent Daily Dose; MDS-UPDRS Movement Disorder Society-Unified Parkinson´s Disease Rating Scale; Me median; MMSE mini mental state examination; PD-CRS Parkinson’s Disease Cognitive Rating Scale; SD standard deviation

^a, b^ Different letters indicate significant differences between groups (*p* < 0.05), and equal letters indicate no differences

**Fig. 1:**
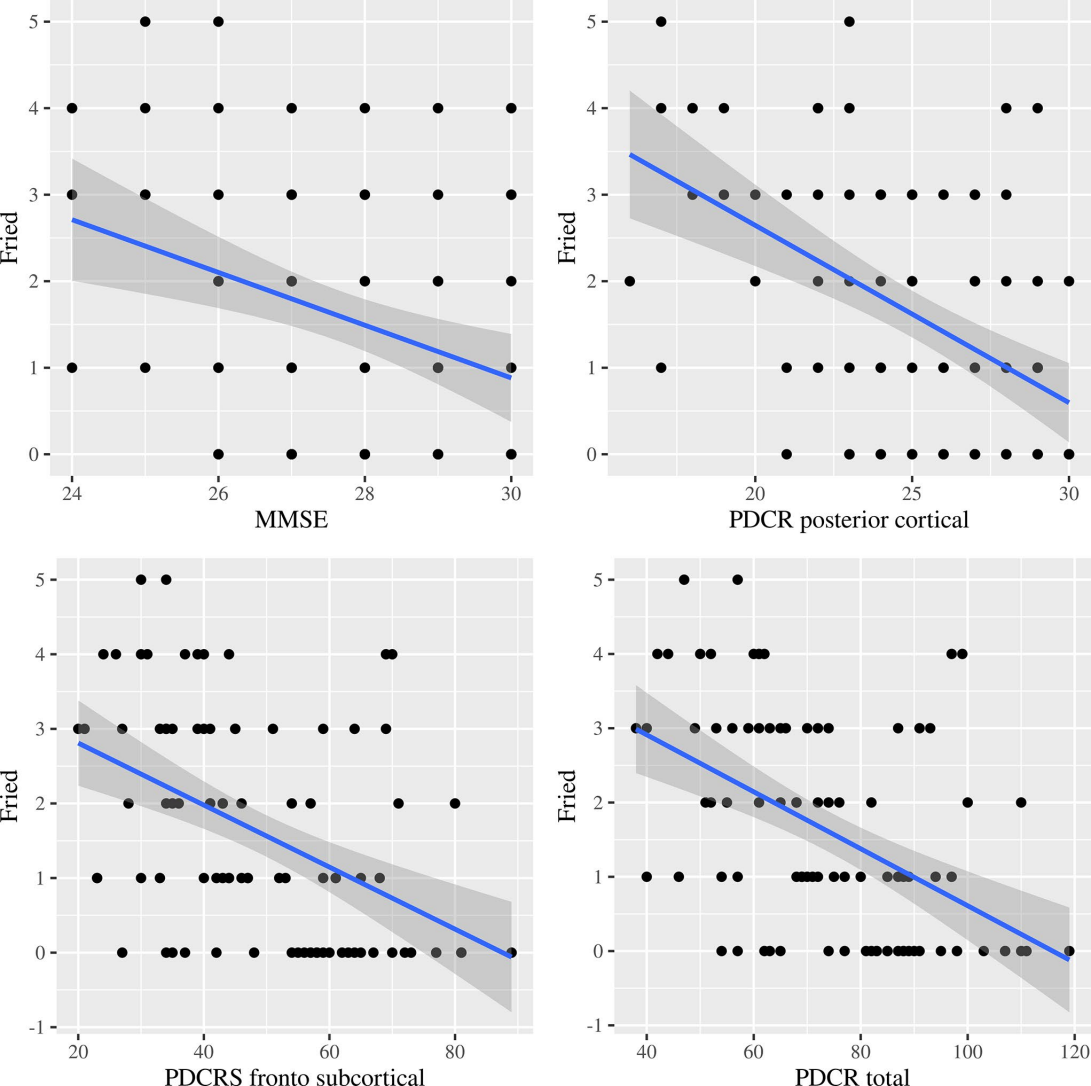
Scatterplot between Fried and MMSE and dimensions of PD-CRS

**Fig. 2:**
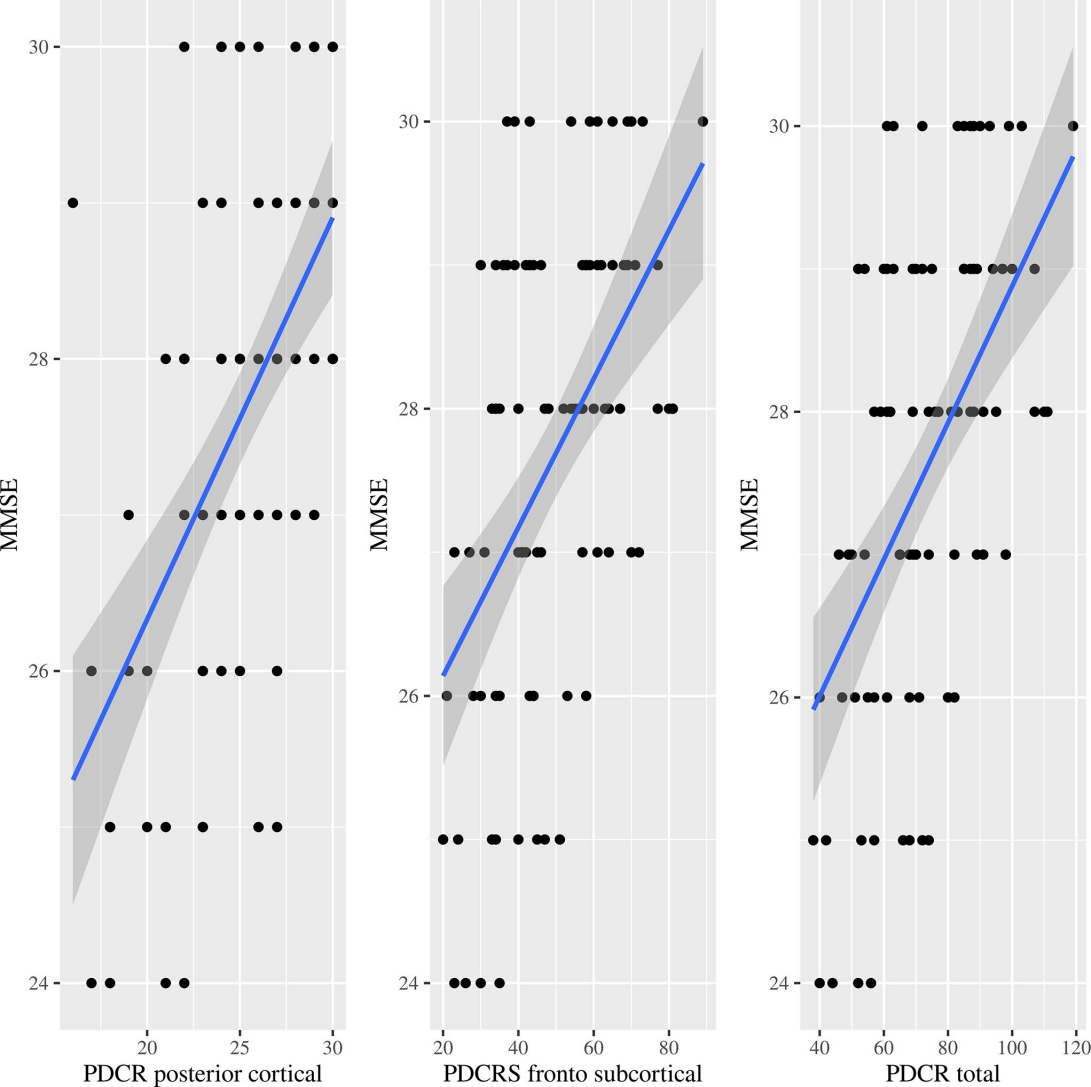
Scatterplot between MMSE and dimensions of PD-CRS

Regarding the markers of frailty, there was a predominance of a decrease in grip strength (= 41, 45.6%), followed by a decrease in walking speed (*n* = 37, 41.1%), low level of physical activity (*n* = 35, 38.9%), exhaustion (*n* = 20, 22.2%), and unintentional weight loss (*n* = 13, 14.4%). Patients with the positive Fried criteria physical activity, walk time and grip strength were statistically and significantly associated (*p* < 0.05) with worse cognitive performance on the MMSE and PD-CRS scales. Thus, the data revealed a statistically significant negative correlation between frailty and measures of patients’ cognitive function. The correlation coefficients for the Fried scale were as follows: -0.302 with the MMSE (*p* = 0.004), -0.503 with the total score of the PD-CRS scale, -0.475 with the frontal-subcortical subscale, and − 0.463 with the posterior cortical subscale, with a *p* < 0.001 in all cases. Figure 1 shows a scatterplot between the Fried scale and the MMSE and the PD-CRS dimensions. On the other hand, a statistically significant positive correlation was observed between the MMSE and the PD-CRS scale. The correlation coefficients of the MMSE were 0.518 with the total score of the PD-CRS scale, 0.485 with the frontal-subcortical subscale, and 0.531 with the posterior cortical subscale, with a p value < 0.001 in all cases. Figure 2 shows another scatterplot between the MMSE and PD-CRS dimensions.

Table 3 shows a multinomial logistic regression model carried out to predict frailty status (frail or prefrail) as a function of several independent variables, such as PD-CRS total score, gender, age, and educational level. The results of this analysis show that the PD-CRS total score has a significant impact on frailty in both groups (frail and pre-frail), in such a way that, the higher the score on this scale, the lower the probability of being pre-frail or frail. For frail patients, gender also plays a significant role, with women being significantly more likely to be frail than men. The variables of age and educational level did not appear to have a significant effect on frailty in these groups.

**Table 3:** Multinomial logistic regression model for frailty

		Coefficient	Significance	Exponential coefficient	IC (95%)
Frail	PD-CRS total	-0.043	**0.038**	0.958	(0.919–0.998)
	Gender man (ref)	-			
	Gender woman	0.506	0.411	1.658	(0.496–5.546)
	Age < 70 (ref)	-			
	Age ≥ 70	0.211	0.770	1.235	(0.299–5.098)
	No education	-			
	Completed primary education	0.002	0.998	1.002	(0.168–5.966)
	Secondary education	-0.559	0.549	0.572	(0.092–3.564)
	University	-0.964	0.307	0.086	(0.060–2.421)
Prefrail	PD-CRS total	-0.081	**0.002**	0.922	(0.877–0.969)
	Gender man (ref)	-			
	Gender woman	1.531	**0.033**	4.621	(1.13-18.868)
	Age < 70 (ref)	-			
	Age ≥ 70	-0.197	0.836	0.821	(0.127–5.288)
	No education	-			
	Completed primary education	-0.503	0.595	0.605	(0.095–3.859)
	Secondary education	-1.295	0.219	0.274	(0.035–2.164)
	University	-2.449	0.067	0.086	(0.006–1.186)

*Abbreviations* IC Confidence interval; Ref Reference; PD-CRS Parkinson’s Disease Cognitive Rating Scale

In addition, Supplementary Table 1 shows the sociodemographic and clinical characteristics distributed by gender. Supplementary Table 2 shows the scores obtained by the patients according to the frailty phenotype for each of the PD-CRS scale tests.

## Discussion

The objective of this study was to analyze the relationship between frailty and cognitive impairment in pwPD. The analysis of our data shows a negative correlation between these two measures, which means that frail patients obtain worse scores on the scales that evaluate cognitive function. These results are in line with those observed by other studies showing a link between frailty and cognitive impairment among pwPD [40, 41]. To our knowledge, the current study is the first to use the PD-CRS scale to distinguish pwPD as robust, pre-frail, and frail. The PD-CRS scale was designed to capture the full spectrum of cognitive deficits associated with PD [39]. Frail pwPD exhibited an impairment of cognitive functions dependent on cortical regions and subcortical structures, these regions being more preserved in the case of robust pwPD. The profile of neuropsychological alteration in pwPD with cognitive alterations is characterized by a fronto-subcortical pattern with alterations in attention and executive functions, accompanied by alterations in memory, visuospatial, visuoperceptual and semantic fluency skills, which suggests a localization of posterior cortical areas [42].

The results of this study showed differences in the cognitive profiles defined by the PD-CRS between robust, prefrail, and frail patients, suggesting that frailty is associated with differential cognitive impairment. In particular, the scores on the subscales highlight two profiles: the frontal-subcortical profile, with greater impairment in frail patients, and the posterior cortical profile, which is less pronounced but also affected by increased frailty. The progression of PD shows distinctive features according to the frailty phenotype. For their part, cognitive and frailty assessments could show a relationship between robust phenotype and better cognitive performance according to PD-CRS. As for the prefrailty phenotype, it would be associated with subtle cognitive changes and early signs of fronto-subcortical dysfunction. As for the frailty phenotype, significant cognitive impairment is observed, presenting alterations in both PD-CRS profiles, as well as a higher burden of motor symptoms. These phenotypic differences have important clinical implications, as they allow for a more personalized approach in the management of pwPD and frailty. Assessing the subscales of the PD-CRS, beyond the total score, helps to identify early patterns of cognitive decline in frail and pre-frail patients, promoting timely interventions. Determining the type of cognitive impairment (fronto-subcortical or posterior cortical) makes it possible to guide the approach to cognitive rehabilitation, predict the potential response to dopaminergic medications, and serve as a prognostic indicator to assess the progression towards dementia [39].

Our data revealed that cognitive function, corresponding to the posterior cortical and fronto-subcortical areas, was more affected in frail patients, translating into lower scores in the tests of: sustained attention, working memory, drawing a clock, delayed free recall verbal memory, alternating verbal fluency, action verbal fluency, confrontation naming and copying a clock. Similar results were obtained in the study by Lin et al. [43]in which frail pwPD showed significantly worse performance in attention, executive function, memory, speech and language, and visuospatial functions.

This association between frailty and cognitive impairment has also been found in older adult populations [44, 45]. Compared to robust patients, frail patients exhibit significantly lower scores in several cognitive domains such as visuospatial function, executive function, sustained attention, working memory, verbal fluency and processing speed [45–47].

The mechanism underlying the pathophysiological foundation of the connection between frailty and cognitive impairment remains unclear and may involve multiple factors [48]. It is very likely that the pathophysiological mechanisms of both conditions overlap for the most part and develop a positive feedback loop that leads to greater frailty and cognitive impairment [48]. It is believed that impaired vascular functions, reduced levels of androgen hormones, low levels of vitamin D and B12, chronic inflammation, insulin resistance, mitochondrial dysfunction, oxidative stress, dysfunction of energy homeostasis, and genetic background may be behind this link [49, 50].

In our study, frail patients were older, probably because the passage of time leads to progressive degeneration at the neuronal level, which conditions the state of fragility. This result is similar to that obtained by other cross-sectional studies [43, 51]involving pwPD and using the Fried phenotype [12]. Likewise, although frailty can occur at any time during adulthood, it has also been associated with an increase in age in the general population [30]. The same happens with PD, which becomes more prevalent as patients age [52]. Both concepts are, therefore, linked to age.

Regarding gender, although there were no differences in frailty status between men and women, our multinomial regression analysis showed that women were more likely to develop frailty. Our data align with those observed in other observational studies [53, 54], in which the female gender is associated with the state of frailty in pwPD when using the Fried phenotype [55]. However, our results may have been conditioned by a sample with a reduced number of women compared to men, which may limit the influence of gender on frailty.

The presence of comorbidities according to the Charlson Index was not representative in this sample, as the values of this index ranged between 0 and 1 points, with no statistically significant differences observed among the three groups of patients categorized according to Fried’s criteria. However, it is worth noting that frail patients did present slightly higher scores. Other authors who assessed frailty using the Clinical Frailty Scale [56] have found a statistically significant association between frailty and a higher Charlson comorbidity index [19]. The presence of three or more comorbidities has been described as a risk factor for frailty in older adults, according to Fried et al. [12], suggesting a degree of reciprocity and influence between the two concepts. Therefore, the presence of frailty could predispose individuals to a higher number of comorbidities. The characteristics of the sample in this study, with an absence of severe illnesses, likely resulted in lower Charlson Index scores across the three groups, which may have influenced the lack of association between frailty and comorbidity.

Disease status and severity were associated with frail patients. Patients with a higher H&Y stage or a higher MDS-UPDRS score were frail and there were statistically significant differences between the groups. These results coincide with those obtained in other studies [51, 54, 57] using the Fried phenotype [12]. A higher stage of the disease or a greater severity of it leads to greater neuronal involvement, which conditions greater physical and functional deterioration, which will be reflected in more positive Fried criteria [12].

Antiparkinsonian medication was not a determinant of frailty status, given that there were no statistically significant differences in LEDD between groups. Despite this, robust patients had a lower LEDD in our study. These data differ from those obtained by Ozër et al. [58]and Lin et al. [43]in which LEDD was associated with frail patients. In the case of Ozër et al. [58]patients were classified according to their dose of levodopa, distinguishing between those taking an amount greater than or equal to 400 mg or less than 400 mg, and not according to the LEDD, like our study and that of Lin et al. [43].

In our study, the tool used to evaluate frailty was the Fried scale, which was chosen because it was the most used in older adults and in pwPD [15, 41]. Despite this, it was not originally a scale designed to detect frailty in pwPD. Most of Fried’s criteria focus on the physical condition. However, the heterogeneity of PD requires a multidimensional approach that includes motor and non-motor criteria, which characterize this disease.

The coexistence of frailty and cognitive impairment should be assessed, as PD patients with both conditions are more vulnerable and have a higher chance of experiencing adverse effects. In this regard, the approach to this patient profile will have to be different. We need to delve into the importance of early detection in both cases, to carry out interventions that are adapted to this type of patient and that, given their dynamic condition, can reverse frailty. The neurodegenerative nature of PD means that it’s important to consider the possibility of patients with cognitive impairment developing dementia. Different authors signal the existence of frailty and cognitive impairment as a risk factor for dementia in older adults [59, 60], some even highlight a greater probability of frail patients with PD developing dementia [29]. Carrying out an intervention to reduce frailty may become a promising tool to be used as a preventive measure for dementia [60]. In this regard, it is necessary to develop early detection programs that evaluate both frailty and cognitive impairment among pwPD, to subsequently implement strategic intervention plans focused on reversing frailty and cognitive deterioration to the extent of the patient’s possibilities. In this way, we can try to improve their health, reduce their vulnerability and the future costs involved in the care and attention of this patient profile. More studies are needed in pwPD pre-frail and frail patients, to evaluate the effects that some common treatment therapies, such as physiotherapy, may have on frailty and their possible influence on preventing the development of dementia.

### Limitations

The limitations of the present study focused on the one hand, on its design, given that its cross-sectional nature does not allow establishing a cause-effect relationship. As for other possible limitations, there is information bias. To minimize this, validated questionnaires translated into Spanish were used and simple and understandable language was used with the patients. Furthermore, memory bias could have been present, given the profile of the patients who were part of the study, so, when necessary, data were completed with the help of the main caregiver.

While we recognize that depression is highly prevalent in PD and significantly impacts cognitive function, as well as potentially contributing to the onset of frailty through symptoms such as fatigue, the focus of our study was specifically on the association between frailty, defined according to Fried’s phenotype, and cognitive impairment. In this study, we deliberately focused on a more specific exploration of frailty through its physical and functional components, which is aligned with the operational criteria of Fried’s model. With regard to CES-D, in this study we employed it only to define the “exhaustion” criterion of Fried’s phenotype. This was done to maintain methodological consistency with Fried’s model and not as a comprehensive assessment of depression or its potential impact on cognitive performance.

Our primary objective was to explore frailty as a physical syndrome, as conceptualized by Fried et al. While depression and fatigue are interrelated, the “exhaustion” criterion in this model does not specifically require a clinical diagnosis or a full assessment of depressive symptoms, but serves as an indicator of a broader physiological and psychological construct. We fully concur that depression is a critical factor that deserves further attention in this population. Future studies could adopt a multidimensional framework for the assessment of frailty, including psychosocial factors, such as depression and anxiety, in addition to physical components. The possible mediating or moderating role of depression in the relationship between frailty and cognitive impairment in PD patients could be examined, as well as exploring interventions targeting depressive symptoms and their impact on the progression of frailty and cognitive impairment in PD patients.

### Strengths

The evaluation of cognitive function was carried out using two scales, the MMSE and the PD-CRS. The latter covers several domains of cognitive functioning specifically for pwPD. The patients were evaluated in an environment known to them, which prevents the environment from negatively influencing the performance on the tests and thus obtaining more realistic data on usual functioning.

## Conclusions

To summarize, pwPD exhibited worse cognitive performance in cognitive function tests assessed using the MMSE and PD-CRS scales. PwPD also showed worse performance in fronto-subcortical and posterior cortical functions. In turn, frail patients were characterized by having higher H&Y stages, being older, and a more advanced severity of the disease.

## Electronic supplementary material

Below is the link to the electronic supplementary material.

Supplementary Material 1

## Data Availability

No datasets were generated or analysed during the current study.
