# Supplementary material for: Associations between frailty and cognitive impairment in Parkinson´s disease: a cross-sectional study
Source: Aging Clin Exp Res. 2025 Jan 3;37(1):19. doi: 10.1007/s40520-024-02922-4 (PMC11698760; doi:10.1007/s40520-024-02922-4)
Supplement: Supplementary file 1 — Supplementary Material 1 [file 40520_2024_2922_MOESM1_ESM.pdf]

TITLE

Associations between frailty and cognitive impairment in Parkinson´s disease: a cross-sectional study.

JOURNAL NAME

Ageing Clinical and Experimental Research

AUTHORS INFORMATION AND FULL AFFILIATIONS

Sousa-Fraguas MC, MD [1,2,3,4*]

[1] University of Oviedo, Department of Surgery and Medical-Surgical Specialties, Faculty of Medicine and Health Sciences, 33006 Oviedo, Spain.

[2] Corporación Fisiogestión, 33202 Gijón, Spain.

[3] Instituto de Neurociencias del Principado de Asturias (INEUROPA), University of Oviedo, 33003 Oviedo, Spain.

[4] Instituto de Investigación Sanitaria del Principado de Asturias (ISPA), 33006 Oviedo, Spain.

Rodríguez-Fuentes G, PhD [5,6]

[5] Universidade de Vigo, Department of Functional Biology and Health Sciences, Faculty of Physiotherapy, 36005 Pontevedra, Spain.

[6] HealthyFit Research Group, Galicia Sur Health Research Institute (IIS Galicia Sur). SERGAS-UVIGO.

Lastra-Barreira D, MD [7]

[7] Jove Hospital Foundation, 33290 Gijón, Spain.

Conejo NM, PhD[3,4,8]

[3] Instituto de Neurociencias del Principado de Asturias (INEUROPA), University of Oviedo, 33003 Oviedo, Spain.

[4] Instituto de Investigación Sanitaria del Principado de Asturias (ISPA), 33006 Oviedo, Spain.

[8] Laboratory of Neuroscience, Department of Psychology, University of Oviedo, 33003 Oviedo, Spain. Neurocon Research Group. University of Oviedo, 33003, Oviedo, Spain.

CORRESPONDING AUTHOR

Correspondence to: Sousa-Fraguas MC. e-mail: sousamaria@uniovi.es

Supplementary Table 1: Sociodemographic and clinical characteristics distributed by sex.

Patient characteristics	Male (n=54)	Female(n=36)	p-value
Age, mean (SD)	73.76 (7.16)	73.11 (6.06)	0.656
Range age years			0.787
50-59, n (%)	3 (75%)	1 (25%)	
60-69, n (%)	8 (50%)	8 (50%)	
70-79, n (%)	32 (61.54%)	20 (38.46%)	
≥80, n (%)	11 (61.11%)	7 (38.89%)	
Level of education			0.463
No education, n (%)	15 (65.22%)	8 (34.78%)	
Completed primary education, n (%)	14 (50%)	14 (50%)	
Secondary education, n (%)	14 (58.33%)	10 (41.67%)	
University, n (%)	11 (73.33%)	4 (26.67%)	
Marital status			0.175
Single, n (%)	2 (50%)	2 (50%)	
Married, n (%)	45 (65.22%)	24 (34.78%)	
Divorced, n (%)	2 (66.67%)	1 (33.33%)	
Widowed, n (%)	5 (35.71%)	9 (64.29%)	
Charlson index, mean (SD)	0.59 (0.88)	0.56 (0.73)	0.835
Disease duration (years), mean (SD)	7.73 (5.63)	7.97 (7.23)	0.86
Hoenh & Yarh, mean (SD)	2 (0.67)	2 (0.77)	0.928
Body mass index, mean (SD)	27.59 (4.18)	27.50 (5.81)	0.937
LEDD, mean (SD)	787.52 (391.29)	974.37 (618.63)	0.113
MDS-UPDRS Total, mean (SD)	52.89 (20.65)	52.75 (21.62)	0.976
MDS-UPDRS part I, mean (SD)	13.63 (5.87)	15.11 (6.77)	0.273
MDS-UPDRS part II, mean (SD)	16.28 (6.37)	15.39 (7.58)	0.55
MDS-UPDRS part III, mean (SD)	19.13 (9.71)	18.72 (10.12)	0.848
MDS-UPDRS part IV, mean (SD)	3.85 (3.59)	3.53 (3.55)	0.675
MMSE, mean (SD)	27.61 (1.69)	27.69 (1.67)	0.818
PD-CRS total, mean (SD)	72.80 (18,96)	76.28 (18.81)	0.394
PD-CRS frontal-subcortical, mean (SD)	47.59 (16.41)	51.33 (15.99)	0.287
PD-CRS posterior-cortical, mean (SD)	25.20 (3.37)	24.94 (3.98)	0.74
Abbreviations: LEDD Levodopa Equivalent Daily Dose; MDS-UPDRS Movement Disorder Society-Unified Parkinson´s Disease Rating Scale; MMSE mini mental state examination; PD-CRS Parkinson’s Disease Cognitive Rating Scale; SD standard deviation.			

Supplementary Table 2: Fried scale and items Parkinson's Disease Cognitive Rating Scale.

PD-CRS	Robust (n=29)	Pre-frail (n=33)	Frailty (n=28)	p-value
Item 1, inmediate free recall verbal memory Me (IQR)	8 (7-10)	8 (7-9)	7 (7-9)	p=0.19
Item 2, confrontation naming, Me (IQR)	18 (17-19)[a]	18 (16-19)[b,a]	16.50 (15-18.25)[b]	**p=0.041**
Item 3, sustained attention Me (IQR)	9 (9-10)[a]	8 (7-9)[b]	5.5 (3.75-8)[b]	**p<0.001**
Item 4, working memory, Me (IQR)	6 (5-7)	5 (3-7)	4 (2-4)	**p<0.001**
Item 5, drawing of a clock, Me (IQR)	7 (6-9)[a]	6 (4-6)[b]	4.5 (3-7.25)[b]	**p=0.003**
Item 6, copy drawing of a clock, Me (IQR)	9 (9-10)	9 (8-9)	7 (5-8)	**p<0.001**
Item 7, delayed free recall verbal memory, mean (SD)	6.38 (2.65)[a]	5.18 (2.52)[a,b]	4.71 (2.54)[b]	**p=0.045**
Item 8, alternating verbal fluency, Me (IQR)	10 (8-14)[a]	8 (6-12)[a]	6 (4-9)[b]	**p<0.001**
Item 9, action verbal fluency, Me (IQR)	12 (8-18)[a]	6 (5-10)[b]	6 (4.75-9)[b]	**p=0.003**

Abbreviations: IQR interquartile range; Me median; PD-CRS Parkinson's Disease Cognitive Rating Scale; SD standard deviation.

[a, b] Different letters indicate significant differences between groups ($p < 0.05$), and equal letters indicate no differences.
